# Co_3_O_4_ Nanoparticles Synthesized With *Urtica dioica* Extract Versus Chemical Reduction: Enhanced ROS Generation, Cytotoxicity, and Cancer‐Cell Selectivity

**DOI:** 10.1155/bca/5365097

**Published:** 2026-11-10

**Authors:** Bilsen Tural, Erdal Ertaş, Ömer Erdoğan, Servet Tural

**Affiliations:** ^1^ Department of Nanotechnology Graduate School of Natural and Applied Sciences Dicle University 21280 Diyarbakir Türkiye dicle.edu.tr; ^2^ Department of Chemistry Education Ziya Gökalp Faculty of Education Dicle University 21280 Diyarbakir Türkiye dicle.edu.tr; ^3^ Department of Food Processing Technical Sciences Vocational School Batman University 72060 Batman Türkiye batman.edu.tr; ^4^ Department of Biochemistry School of Medicine Gaziantep Islam Science and Technology University 27010 Gaziantep Türkiye

**Keywords:** anticancer activity, cobalt oxide nanoparticles, green synthesis, reactive oxygen species, selectivity index, *Urtica dioica*

## Abstract

Cobalt oxide (Co_3_O_4_) nanoparticles are redox‐active metal oxides with promising anticancer activity, but whether *Urtica dioica* extract‐mediated synthesis improves their performance relative to conventional chemical synthesis remains unclear. In this study, bare Co_3_O_4_ nanoparticles were prepared by chemical reduction and *Urtica dioica* extract‐synthesized nanoparticles (UD–Co_3_O_4_) were prepared by green synthesis in which the extract constituted the reaction medium, and the two formulations were compared. Energy‐dispersive X‐ray spectroscopy and infrared spectroscopy confirmed an *Urtica dioica*‐derived organic coating on UD–Co_3_O_4_, which increased the hydrodynamic size (108.0 ⟶ 169.9 nm) and reduced the magnitude of the surface charge (−27.23 ⟶ −18.19 mV). Cytotoxicity was evaluated against A549, MDA‐MB‐231 and SH‐SY5Y cancer cells and nontumorigenic L929 fibroblasts, with parallel measurement of intracellular reactive oxygen species (ROS). UD–Co_3_O_4_ was several‐fold more cytotoxic than the bare Co_3_O_4_ against all cancer lines, with IC_50_ values of 29.4, 55.1 and 62.6 μg mL^−1^ versus 320.7, 307.1 and > 1000 μg mL^−1^, respectively, corresponding to an approximately 5‐ to > 15‐fold increase in potency. UD–Co_3_O_4_ also generated higher ROS (for example, 324% vs. 279% of control in A549 at 1000 μg mL^−1^). Crucially, UD–Co_3_O_4_ showed cancer selectivity (selectivity index of 1.94 in A549 and 1.04 in MDA‐MB‐231) that the bare Co_3_O_4_ lacked (SI ≤ 0.28). ROS was the strongest predictor of viability in random‐forest analysis (relative importance 0.58), and analysis of covariance indicated significantly greater cytotoxicity per unit of oxidative stress in cancer cells (pooled *p* = 0.007). *Urtica dioica* extract synthesis therefore enhances both the potency and the cancer selectivity of Co_3_O_4_ nanoparticles, consistent with a ROS‐mediated mechanism.

## 1. Introduction

Cancer remains one of the leading causes of death worldwide, with an estimated 20 million new cases and 9.7 million deaths recorded in 2022 and a burden that continues to rise [1, 2]. Conventional chemotherapy, although effective, is constrained by dose‐limiting systemic toxicity, poor tumor selectivity and the emergence of multidrug resistance, which collectively narrow the therapeutic window and motivate the search for more selective treatment modalities [3]. Nanomedicine has emerged as a powerful framework to address these limitations, offering improved bioavailability, tunable physicochemical properties and the capacity to discriminate between malignant and healthy cells through differences in their microenvironment and redox state [4].

Among nanoscale agents, metal oxide nanoparticles have attracted particular attention because several of them are intrinsically cytotoxic to cancer cells while sparing their normal counterparts, an activity frequently attributed to the generation of reactive oxygen species (ROS) and the induction of apoptosis or necrosis [5]. Zinc, copper, yttrium, and cobalt oxides have all been reported to kill tumor cells through oxidative mechanisms [6–8]. More broadly, redox‐active metal‐based nanomaterials can drive tumor‐cell death through the intracellular release of metal ions and the perturbation of mitochondrial redox homeostasis, further underscoring oxidative stress as a central antitumor mechanism [9]. This selective vulnerability is grounded in cancer redox biology: tumor cells typically sustain elevated basal ROS levels as a consequence of oncogene activation and metabolic reprogramming, and therefore operate closer to the threshold at which further oxidative insult triggers programmed cell death than do normal cells [10, 11]. Nanoparticles that raise the intracellular ROS set‐point above this threshold can thus exert a degree of cancer selectivity [12, 13].

Cobalt oxide (Co_3_O_4_) is a mixed‐valence, redox‐active oxide whose Co(II)/Co(III) couple can participate in Fenton‐type and Haber–Weiss reactions, making it a strong candidate for ROS‐mediated anticancer action [14, 15]. The cytotoxicity of cobalt‐based nanomaterials has been linked to mitochondrial dysfunction, glutathione depletion, lipid peroxidation, and ferroptosis‐like cell death, all of which originate from perturbation of cellular redox homeostasis [8, 16]. As a biologically relevant trace metal that is nevertheless toxic in excess, cobalt sits squarely within the metal‐ion toxicity paradigm in which redox cycling and oxidative stress mediate both therapeutic and adverse effects [17–19].

The route used to prepare metal oxide nanoparticles strongly influences their surface chemistry and biological behavior. Conventional physical and chemical syntheses often rely on hazardous reagents and high energy input, whereas green synthesis using plant extracts provides an eco‐friendly alternative in which phytochemicals act simultaneously as reducing and capping agents [20, 21]. Plant‐mediated Co_3_O_4_ nanoparticles have been obtained from a range of botanical sources and shown to possess photocatalytic, antibacterial, and anticancer activities [22–25]. Recent studies have specifically reported green‐synthesized cobalt oxide nanoparticles with measurable cytotoxicity against human cancer cell lines [26, 27], and comparable plant‐extract‐mediated strategies have been extended to other redox‐active metal‐oxide systems, such as Alchemilla vulgaris‐functionalized Fe_3_O_4_ nanoparticles evaluated against the same panel of cancer and normal cell lines [28], yet direct, quantitative comparisons of green‐synthesized and conventionally synthesized Co_3_O_4_ under identical conditions, together with an explicit assessment of cancer‐cell selectivity, remain scarce. Among the numerous medicinal plants used in green nanotechnology, *Urtica dioica* was selected because it is particularly rich in polyphenols, flavonoids, phenolic acids, carotenoids, sterols, and other redox‐active phytochemicals that can simultaneously act as reducing, complexing, and surface‐capping agents during nanoparticle synthesis [29–31]. These phytochemicals are capable of coordinating cobalt ions and modulating nanoparticle nucleation, growth, colloidal stability, and surface chemistry, while their redox‐active functional groups may also influence nanoparticle–cell interactions and oxidative stress responses [20, 29–31]. Although *Urtica dioica* has been successfully employed for the green synthesis of silver and copper oxide nanoparticles with promising biological activities [32, 33], its application to Co_3_O_4_ nanoparticle synthesis, particularly in studies directly comparing their physicochemical characteristics, ROS‐mediated anticancer activity, and cancer‐cell selectivity with chemically synthesized Co_3_O_4_ prepared under identical experimental conditions, has not been systematically investigated.

A recurring challenge in this field is that surface modification does not always improve performance. Organic capping layers can alter nanoparticle size, dispersion, and surface charge, and may either enhance or attenuate the intrinsic cytotoxicity and selectivity of the inorganic core. Moreover, anticancer potential is meaningful only when accompanied by an acceptable selectivity index (SI) relative to nontumorigenic cells, a parameter that is frequently underreported. Disentangling the contributions of concentration, ROS generation and surface coating to the observed cytotoxicity requires an analysis that goes beyond pairwise comparisons; multivariate and machine‐learning approaches such as principal component analysis (PCA) and random‐forest modeling have recently proven valuable for interpreting such complex nanotoxicological datasets [34–36].

To address these knowledge gaps, the present study directly compared chemically synthesized Co_3_O_4_ nanoparticles with *Urtica dioica*‐mediated Co_3_O_4_ nanoparticles prepared under comparable experimental conditions. The two formulations were comprehensively characterized by FTIR, dynamic light scattering (DLS), zeta‐potential analysis, scanning electron microscopy, and energy‐dispersive X‐ray spectroscopy (EDX). Their anticancer activity was evaluated against three human cancer cell lines (A549 lung adenocarcinoma, MDA‐MB‐231 triple‐negative breast carcinoma, and SH‐SY5Y neuroblastoma) together with the nontumorigenic L929 fibroblast cell line, while intracellular ROS generation was quantified in parallel. By integrating dose–response (IC_50_) and selectivity‐index analyses with two‐way analysis of variance (ANOVA), ROS–viability regression (analysis of covariance [ANCOVA]), and machine‐learning approaches, this study aimed to determine whether *Urtica dioica*‐mediated surface functionalization enhances the ROS‐mediated anticancer activity and cancer‐cell selectivity of Co_3_O_4_ nanoparticles, and to identify the principal physicochemical and biological factors underlying these effects.

## 2. Materials and Methods

### 2.1. Materials

Cobalt(II) chloride hexahydrate (CoCl_2_·6H_2_O) and cobalt(II) nitrate hexahydrate (Co(NO_3_)_2_·6H_2_O) were used as cobalt precursors. Sodium borohydride (NaBH_4_) and citric acid were used as reducing and stabilizing agents, respectively. All chemicals were of analytical grade and used as received without further purification. Deionized water was used throughout all experiments. Fresh leaves of *Urtica dioica* L. were collected in Diyarbakır, Türkiye, in May 2025, and the plant material was taxonomically identified by an expert botanist from the Department of Biology, Dicle University, Diyarbakır, Türkiye. The leaves were washed thoroughly with distilled water to remove surface impurities and air‐dried at room temperature prior to extraction.

### 2.2. Preparation of the *Urtica dioica* Extract

The dried leaves were finely ground, and approximately 10 g of the powdered material was mixed with 100 mL of deionized water. The mixture was heated at 60°C–70°C for 30 min under continuous stirring. After cooling to room temperature, the extract was filtered through Whatman No. 1 filter paper to remove solid residues and stored at 4°C until further use.

### 2.3. Chemical Synthesis of Co_3_O_4_ Nanoparticles

For the synthesis of Co_3_O_4_ nanoparticles, 2.0 g of CoCl_2_·6H_2_O was dissolved in 120 mL of ultrapure water under an argon atmosphere to prevent oxidation and to maintain inert conditions in the reaction medium. After a homogeneous solution had been obtained, 0.2 g of citric acid was added to control particle formation and prevent agglomeration, and the mixture was stirred magnetically at room temperature for 30 min. A total of 200 mL of 0.8 M NaBH_4_ solution was then added dropwise under continuous stirring while the argon atmosphere was maintained. The appearance of black particles upon the onset of NaBH_4_ addition indicated the beginning of nanoparticle formation. After the addition of the reducing agent was complete, the reaction mixture was stirred for a further 30 min at room temperature under argon to allow the reaction to reach completion. The resulting nanoparticles were separated from the reaction medium with a neodymium magnet and washed five times with deionized water to remove unreacted reagents and by‐products from the surface. Finally, the purified nanoparticles were freeze‐dried, and the resulting Co_3_O_4_ nanoparticles were stored at room temperature in the dark until characterization and application studies.

### 2.4. Green Synthesis of UD–Co_3_O_4_ Nanoparticles

For the green synthesis of UD–Co_3_O_4_ nanoparticles, 10 mL of an aqueous Co(NO_3_)_2_·6H_2_O solution containing 2.0 g of the cobalt precursor was added to 100 mL of *Urtica dioica* extract under continuous stirring. The pH of the mixture was adjusted to 9.0 using an NH_4_OH solution. The reaction mixture was then stirred at 600 rpm and maintained at 80°C for 2 h to promote the formation and precipitation of cobalt oxide nanoparticles.

After completion of the reaction, the resulting precipitate was separated from the reaction medium and washed three times with deionized water to remove unreacted precursors, residual extract components, and other impurities. The washed nanoparticles were subsequently dispersed in 50 mL of fresh *Urtica dioica* extract and stirred at room temperature for 30 min to enhance the adsorption of phytochemical constituents onto the nanoparticle surface. The particles were then separated, washed again with deionized water to remove unbound phytochemicals, and freeze‐dried. The resulting UD–Co_3_O_4_ nanoparticles were stored in the dark at room temperature until further characterization and application studies [37, 38].

### 2.5. Characterization

The morphology and surface structure of the two formulations were examined by scanning electron microscopy (SEM; GeminiSEM 300, Zeiss GmbH, Oberkochen, Germany) operated at an accelerating voltage of 30.00 kV and a magnification of 100,000×, and their elemental composition was determined by EDX. The hydrodynamic size and polydispersity index (PDI) were measured by DLS, and the zeta potential was determined by electrophoretic light scattering, both using a Malvern Zetasizer Nano ZS (Malvern Instruments, Malvern, UK) at 25°C; particle size distributions are reported as intensity‐weighted distributions with Z‐average (intensity‐weighted mean) hydrodynamic diameters. Fourier‐transform infrared spectroscopy (FTIR) analysis was carried out using an ATR‐equipped FTIR spectrometer (PerkinElmer Spectrum Two, PerkinElmer Inc., Waltham, MA, USA) within the spectral range of 4000–400 cm^−1^. The obtained spectra were used to identify the functional groups present in the plant extract and synthesized nanoparticles, as well as to investigate the role of phytochemicals in nanoparticle synthesis and surface stabilization.

### 2.6. Cell Culture

Human lung adenocarcinoma (A549; human, male), human triple‐negative breast carcinoma (MDA‐MB‐231; human, female) and human neuroblastoma (SH‐SY5Y; human, female) cell lines were used as cancer models, and the mouse subcutaneous fibroblast line (L929; mouse, male) was used as a nontumorigenic reference. The L929 line was chosen as the normal‐cell reference because it is the fibroblast line recommended in the ISO 10993‐5 standard for the in vitro cytotoxicity testing of biomaterials and is very widely used for this purpose, which makes the selectivity indices reported here directly comparable with the large body of nanomaterial cytotoxicity literature that adopts the same reference. All cell lines were obtained from the American Type Culture Collection (ATCC, Manassas, VA, USA), where cell line identity had been authenticated by short tandem repeat (STR) profiling. Upon receipt, cells were expanded and maintained according to ATCC recommendations. Cultures were routinely monitored for mycoplasma contamination using a PCR‐based detection assay, and only mycoplasma‐free cells were used in the experiments. Cells were cultured in Dulbecco’s Modified Eagle Medium (DMEM) supplemented with 10% (v/v) fetal bovine serum (FBS) and 1% (v/v) penicillin–streptomycin, and maintained at 37°C in a humidified atmosphere containing 5% CO_2_. Cells were subcultured at 70%–80% confluency using standard trypsinization.

### 2.7. Cell Viability Assay

Cell viability was assessed by the MTT assay. Cells were seeded in 96‐well plates at a density of 1 × 10^4^ cells per well and allowed to adhere for 24 h. They were then treated for 24 h with Co_3_O_4_ or UD–Co_3_O_4_ nanoparticles at 1, 10, 100, and 1000 μg mL^−1^, with untreated cells serving as the control. After treatment, MTT solution (5 mg mL^−1^) was added to each well and incubated for 3–4 h at 37°C; the resulting formazan crystals were dissolved in dimethyl sulfoxide (DMSO), and the absorbance was read at 570 nm on a microplate reader. Viability was expressed as a percentage of the untreated control. For each treatment group, the assay was performed in technical triplicate (three replicate wells within the same experimental plate), and the reported values represent the mean of these three technical replicates.

### 2.8. IC_50_ Determination and SI

Half‐maximal inhibitory concentration (IC_50_) values were obtained from the dose–response data by nonlinear regression using a four‐parameter logistic (4PL) model fitted to log_10_‐transformed concentrations, with the upper and lower asymptotes constrained to 100% and 0%, respectively. Goodness of fit was evaluated by the coefficient of determination (*R* ^2^), and 95% confidence intervals and Hill slopes were recorded for each fit. The SI was calculated as the ratio of the IC_50_ in the nontumorigenic L929 line to that in each cancer line:

(1) SI= IC50L929 IC50cancer line,

where SI > 1 indicates preferential cytotoxicity toward the cancer line. When the IC_50_ exceeded the highest tested concentration (1000 μg mL^−1^), the value was reported as not determined (n.d.).

### 2.9. Measurement of Intracellular ROS

Intracellular ROS were measured using the 2′,7′‐dichlorofluorescin diacetate (DCFH‐DA) assay. Cells were seeded in 96‐well plates and allowed to attach overnight, then treated for 24 h with Co_3_O_4_ or UD–Co_3_O_4_ nanoparticles at 1, 10, 100, and 1000 μg mL^−1^, with untreated cells as the control. After treatment, the cells were washed with phosphate‐buffered saline (PBS) to remove excess nanoparticles and incubated with 10 μM DCFH‐DA at 37°C for 30 min in the dark; excess dye was then removed by washing with PBS. Fluorescence was recorded on a microplate reader at excitation and emission wavelengths of 485 and 528 nm, respectively, and ROS levels were expressed as a percentage of the untreated control. As for the viability assay, ROS measurements were performed in technical triplicate (three replicate wells within the same experimental plate) for each treatment group, and the reported values represent the mean of these three technical replicates.

### 2.10. Statistical and Multivariate Analysis

Data are presented as mean ± standard deviation (SD) of three technical replicates (triplicate wells within the same plate). Differences between the two formulations at each concentration were assessed by Welch’s t‐test, and the combined effects of formulation and concentration on cell viability were evaluated for each cell line by two‐way ANOVA. The relationship between ROS generation and cell viability was examined by linear regression, and the slopes obtained for the two formulations were compared by ANCOVA. To identify the principal determinants of cytotoxicity across the full dataset, PCA and random‐forest regression (with concentration, ROS level, and nanoparticle type as predictors of viability) were performed. The random‐forest model (100 trees, fixed random seed of 42) was trained on all 96 observations (four cell lines, two formulations, four concentrations and three replicates), and its predictive performance was assessed by two complementary cross‐validation schemes: a standard fivefold cross‐validation and a more conservative leave‐one‐cell‐line‐out cross‐validation, in which all data from one cell line were held out in turn to test generalization to an unseen cell line. Feature‐importance stability was examined by recomputing the importance ranking within each cross‐validation fold. A value of *p* < 0.05 was considered statistically significant. Curve fitting, statistical testing, and multivariate analyses were carried out in Python (SciPy, scikit‐learn and statsmodels libraries).

### 2.11. Use of Large Language Models

A generative AI tool (a large language model) was used solely for language editing and to assist in drafting scripts for data visualization and statistical analysis, which were subsequently verified, tested, and executed by the authors. The tool was not used to generate, modify, analyze, or interpret any experimental data, results, or scientific conclusions. All analyses were performed on the authors’ own experimental data using standard, auditable scientific software (Python: SciPy, scikit‐learn, statsmodels), and all statistical outputs and figures were independently reproduced and verified by the authors from the raw data. The authors take full responsibility for the accuracy and integrity of all content, in accordance with the journal’s authorship policy.

## 3. Results

### 3.1. Morphological and Elemental Characterization

SEM imaging showed that both formulations consisted of aggregated, near‐spherical nanoparticles (Figure 1a,b), consistent with the aggregated morphologies commonly observed for green‐synthesized metal‐oxide nanoparticles [39]. The morphology of the two samples was broadly similar, although the UD–Co_3_O_4_ particles formed somewhat denser aggregates with a less sharply defined surface, consistent with the presence of an organic surface layer. EDX analysis confirmed the cobalt oxide composition of both samples (Figure 1c,d). The bare Co_3_O_4_ sample contained only oxygen and cobalt (20.08 and 79.92 wt%, respectively), whereas the UD–Co_3_O_4_ sample contained an additional, substantial carbon signal (31.14 wt%) alongside oxygen (11.89 wt%) and cobalt (56.97 wt%). The appearance of this carbon contribution only in the green‐synthesized sample provides direct elemental evidence for the *Urtica dioica*‐derived organic coating on the UD–Co_3_O_4_ nanoparticles.

**FIGURE 1:**
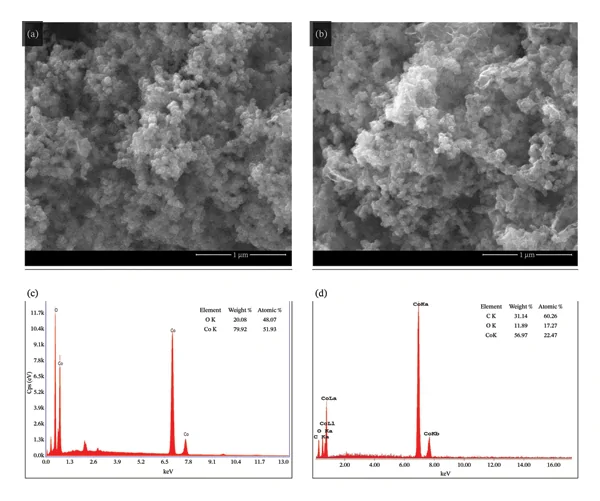
Morphological and elemental characterization of the nanoparticles. (a) SEM micrograph of Co_3_O_4_ NPs and (b) UD–Co_3_O_4_ NPs showing the aggregated, near‐spherical particle morphology; (c) EDX spectrum of Co_3_O_4_ NPs and (d) UD–Co_3_O_4_ NPs. In (a) and (b), the scale bar represents 1 μm. The Co and O signals confirm the cobalt oxide phase, whereas the additional carbon contribution in d is consistent with the *Urtica dioica*‐derived organic surface layer. SEM images were acquired at an accelerating voltage of 30.00 kV and a magnification of 100,000×; the scale bar in (a) and (b) represents 1 μm.

### 3.2. FTIR Analysis

FTIR spectroscopy was used to probe the functional groups associated with the two formulations and the *Urtica dioica* extract (Figure 2). Both nanoparticle samples displayed the characteristic metal–oxygen stretching vibrations of the spinel cobalt oxide lattice in the low‐wavenumber region. Specifically, both samples showed the two characteristic Co–O absorption bands of the spinel Co_3_O_4_ structure below about 700 cm^−1^, corresponding to the stretching vibrations of Co^2+^–O at tetrahedral sites and Co^3+^–O at octahedral sites of the cubic spinel lattice; the simultaneous appearance of these two bands is a well‐established diagnostic fingerprint of phase‐pure Co_3_O_4_ and, together with the EDX composition, confirms the formation of the spinel cobalt oxide phase in both formulations. The UD–Co_3_O_4_ spectrum additionally showed a broad O–H band that was shifted relative to the bare sample, together with C=C and intensified C–O features attributable to phenolic and flavonoid components of the extract. These bands, absent or weak in bare Co_3_O_4_, indicate that phytochemical constituents were adsorbed onto the UD–Co_3_O_4_ surface and are consistent with the carbon signal detected by EDX.

**FIGURE 2:**
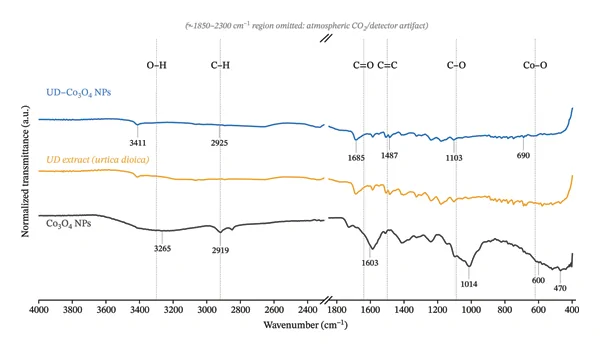
FTIR spectra of Co_3_O_4_ NPs, the *Urtica dioica* (UD) extract and UD–Co_3_O_4_ NPs. Vertical guide lines indicate the principal functional‐group regions (O–H, C–H, C=O, C=C, C–O and Co–O stretching). The shift of the O–H band (3265 ⟶ 3411 cm^−1^) together with the appearance of C=C and intensified C–O bands in UD–Co_3_O_4_ NPs indicates adsorption of phytochemical components on the nanoparticle surface; the ≈1850–2300 cm^−1^ region (atmospheric CO_2_/detector artifact) is omitted from the wavenumber axis.

### 3.3. Hydrodynamic Size and Surface Charge

DLS and zeta‐potential measurements revealed clear differences between the two formulations (Figure 3; Table 1); DLS and zeta‐potential analysis of this type are standard tools for probing the hydrodynamic size, colloidal stability and surface charge of nanoparticle systems [40], including redox‐active metal‐oxide nanostructures [41]. The Z‐average hydrodynamic diameter increased from 108.0 nm for bare Co_3_O_4_ to 169.9 nm for UD–Co_3_O_4_, and the PDI rose from 0.2433 to 0.3797, indicating that surface capping increased both the apparent particle size and the breadth of the size distribution. The zeta potential, which was strongly negative for bare Co_3_O_4_ (−27.23 mV), became less negative for UD–Co_3_O_4_ (−18.19 mV), consistent with partial masking of the oxide surface charge by the adsorbed organic layer. The larger hydrodynamic size measured by DLS relative to the primary particle dimensions seen by SEM reflects aggregation and the hydrated organic shell in suspension [42, 43].

**FIGURE 3:**
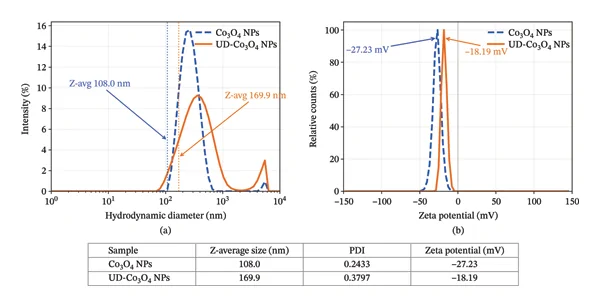
Hydrodynamic size and surface charge of the nanoparticles. (a) Intensity‐weighted hydrodynamic size distributions with Z‐average values indicated and (b) zeta‐potential distributions of Co_3_O_4_ NPs and UD–Co_3_O_4_ NPs. Surface capping by *Urtica dioica* components increased the hydrodynamic diameter (108.0 ⟶ 169.9 nm) and polydispersity index (0.2433 ⟶ 0.3797) and reduced the magnitude of the negative zeta potential (−27.23 ⟶ −18.19 mV).

**TABLE 1:** Physicochemical characterization of Co_3_O_4_ and UD–Co_3_O_4_ nanoparticles.

Sample	Z‐average size (nm)	PDI	Zeta potential (mV)	Morphology
Co_3_O_4_ NPs	108.0	0.2433	−27.23	Aggregated, near‐spherical
UD–Co_3_O_4_ NPs	169.9	0.3797	−18.19	Surface‐modified, quasi‐spherical

*Note:* Z‐average hydrodynamic size, polydispersity index (PDI), and zeta potential were obtained by dynamic light scattering; morphology was assessed by SEM. Principal FTIR assignments are described in the text.

### 3.4. Cytotoxicity and Dose–Response Analysis

The cytotoxicity of the two formulations was assessed by MTT assay against three cancer cell lines (A549, MDA‐MB‐231, and SH‐SY5Y) and the nontumorigenic L929 line over the 1–1000 μg mL^−1^ range (Figure 4). Both formulations reduced cell viability in a concentration‐dependent manner, but UD–Co_3_O_4_ was markedly more cytotoxic than bare Co_3_O_4_ against all three cancer lines at the higher concentrations. In A549 cells, for example, viability at 100 μg mL^−1^ fell to 29.2% with UD–Co_3_O_4_ compared with 60.8% with bare Co_3_O_4_. Two‐way ANOVA confirmed that both formulation and concentration, as well as their interaction, had highly significant effects on viability in every cancer line (*p* < 0.0001; Table S1). In the L929 reference line the effect of formulation was weak (*p* = 0.019) and the interaction term was not significant (*p* = 0.495), indicating that the two formulations behaved similarly in nontumorigenic cells. Pairwise Welch t‐tests at each concentration (Figure 4) showed significant differences between the formulations at most concentrations in the cancer lines but at none in L929.

**FIGURE 4:**
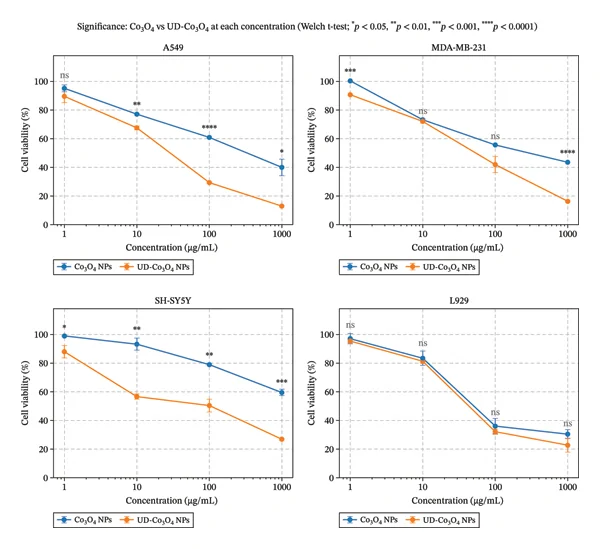
Concentration‐dependent cytotoxicity (MTT assay) of Co_3_O_4_ NPs and UD–Co_3_O_4_ NPs against A549, MDA‐MB‐231, SH‐SY5Y and L929 cells over the 1–1000 μg mL^−1^ range. Data are mean ± SD (*n* = 3). Significance markers denote the Co_3_O_4_ versus UD–Co_3_O_4_ comparison at each concentration (Welch t‐test; ^∗^ *p* < 0.05, ^∗∗^ *p* < 0.01, ^∗∗∗^ *p* < 0.001, ^∗∗∗∗^ *p* < 0.0001; ns not significant). No significant difference was observed at any concentration in the nontumorigenic L929 line.

IC_50_ values derived from 4PL fits (Table 2; Table S2; Figure S1a) quantified this difference. UD–Co_3_O_4_ had substantially lower IC_50_ values than bare Co_3_O_4_ in every cancer line: 29.4 μg mL^−1^ (95% CI 25–35) versus 320.7 μg mL^−1^ (95% CI 221–465) in A549, 55.1 μg mL^−1^ (95% CI 47–65) versus 307.1 μg mL^−1^ (95% CI 150–630) in MDA‐MB‐231, and 62.6 μg mL^−1^ (95% CI 33–118) versus a value exceeding the tested range (> 1000 μg mL^−1^, not determined) in SH‐SY5Y. In the L929 line the two IC_50_ values were closer, at 57.2 μg mL^−1^ (95% CI 36–92) versus 87.3 μg mL^−1^ (95% CI 45–171). The nonoverlapping confidence intervals in the A549 and MDA‐MB‐231 cancer lines underline the markedly higher potency of the green‐synthesized formulation. All fits were of good quality (*R* ^2^ = 0.89–0.99), and the full set of confidence intervals, goodness‐of‐fit values and Hill slopes is provided in Table S2.

**TABLE 2:** IC_50_ values and selectivity indices (SI) of Co_3_O_4_ and UD–Co_3_O_4_ nanoparticles.

Cell line	Co_3_O_4_ IC_50_ (μg mL^−1^)	Co_3_O_4_ SI	UD–Co_3_O_4_ IC_50_ (μg mL^−1^)	UD–Co_3_O_4_ SI
A549	320.7	0.27	29.4	1.94
MDA‐MB‐231	307.1	0.28	55.1	1.04
SH‐SY5Y	> 1000	n.d.	62.6	0.91
L929	87.3	—	57.2	—

*Note:* IC_50_ values were determined by four‐parameter logistic regression on log_10_‐transformed concentrations (top = 100%, bottom = 0%). SI = IC_50_(L929)/IC_50_(cancer line); SI > 1 indicates preferential cytotoxicity toward the cancer line. L929 is the nontumorigenic reference line. n.d. not determined (IC_50_ > 1000 μg mL^−1^).

### 3.5. Cancer‐Cell Selectivity

The SI = IC_50_(L929)/IC_50_(cancer line) showed that surface modification improved cancer selectivity (Table 2; Figure S1b). UD–Co_3_O_4_ exceeded the threshold of preferential cancer toxicity (SI > 1) in A549 (SI = 1.94) and MDA‐MB‐231 (SI = 1.04), and approached it in SH‐SY5Y (SI = 0.91). In contrast, bare Co_3_O_4_ showed no cancer selectivity in any line (SI = 0.27 and 0.28 for A549 and MDA‐MB‐231; not determined for SH‐SY5Y), indicating that the bare Co_3_O_4_ was, if anything, more toxic to the nontumorigenic line than to the cancer lines.

### 3.6. Intracellular ROS Generation

Intracellular ROS, measured by the DCFH‐DA assay, increased with concentration for both formulations in all cell lines (Figure 5). At every matched condition, UD–Co_3_O_4_ elicited higher ROS levels than bare Co_3_O_4_; in A549 cells at 1000 μg mL^−1^, for instance, ROS reached 324% of control for UD–Co_3_O_4_ versus 279% for bare Co_3_O_4_. Pooling the two formulations, ROS generation was strongly and negatively correlated with cell viability in every line (Figure 6; A549 *r* = −0.96; MDA‐MB‐231 *r* = −0.96; SH‐SY5Y *r* = −0.84; L929 *r* = −0.91; all *p* < 0.001; Table S3).

**FIGURE 5:**
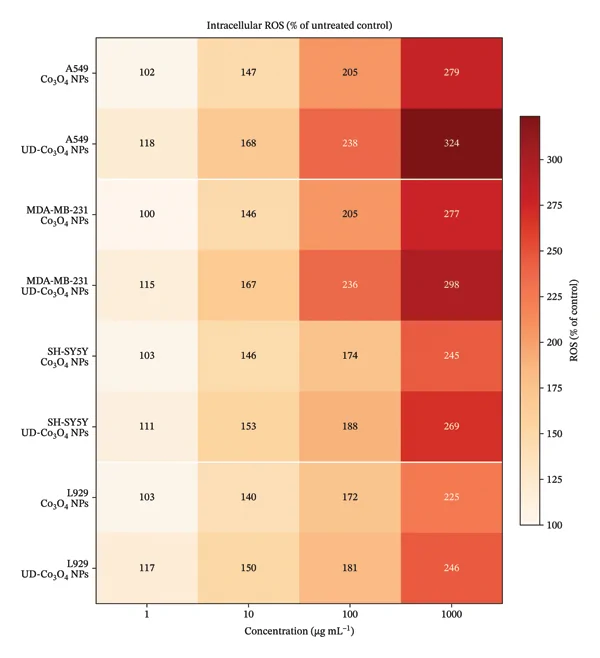
Intracellular reactive oxygen species (ROS) generation expressed as a percentage of the untreated control for each cell line and formulation over the 1–1000 μg mL^−1^ range. ROS levels increased in a concentration‐dependent manner for both formulations in all cell lines.

**FIGURE 6:**
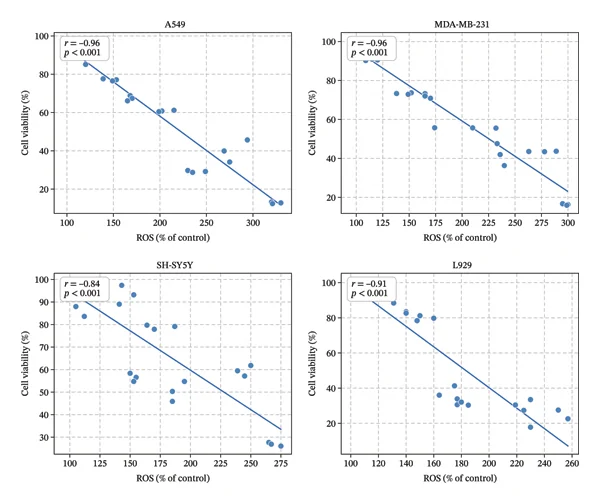
Relationship between intracellular ROS generation and cell viability for each cell line, with Co_3_O_4_ and UD–Co_3_O_4_ data pooled. Solid lines are linear regression fits, and Pearson correlation coefficients are given in each panel (A549 *r* = −0.96; MDA‐MB‐231 *r* = −0.96; SH‐SY5Y *r* = −0.84; L929 *r* = −0.91; all *p* < 0.001).

To compare the efficiency with which ROS translated into loss of viability, the ROS–viability regression slopes of the two formulations were compared by ANCOVA (Table S4). In the cancer lines the slope was significantly steeper for UD–Co_3_O_4_ than for bare Co_3_O_4_ (A549 *p* = 0.029; MDA‐MB‐231 *p* = 0.005), and the same trend was seen for SH‐SY5Y, whereas in L929 the slopes were indistinguishable (*p* = 0.918). Pooled across the three cancer lines the difference was significant (*p* = 0.007), indicating that UD–Co_3_O_4_ produced greater cytotoxicity per unit of ROS in cancer cells specifically.

### 3.7. Multivariate Analysis

PCA of the combined dataset (variables: concentration, ROS and viability) captured 93.6% of the total variance on the first principal component and separated the two formulations primarily along this axis (Figure 7a). Random‐forest regression identified ROS as the strongest predictor of cell viability (relative importance 0.58), followed by concentration (0.36) and nanoparticle type (0.06) (Figure 7b; Table S5), underscoring the central role of oxidative stress in the observed cytotoxicity. Consistent with the ANCOVA, the global ROS–viability relationship was steeper for UD–Co_3_O_4_ (*r* = −0.93) than for bare Co_3_O_4_ (*r* = −0.84) (Figure 7c).

**FIGURE 7:**
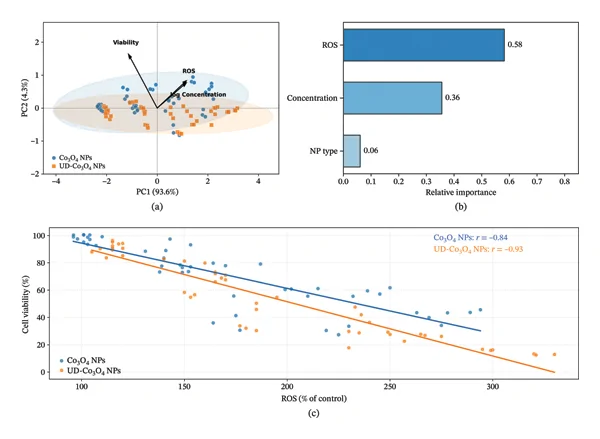
Multivariate analysis of the combined dataset. (a) Principal component analysis biplot (variables: concentration, ROS, viability) with 95% confidence ellipses, showing separation of the two formulations primarily along PC1 (93.6% of the variance); (b) random‐forest relative feature importance for prediction of cell viability (ROS 0.58, concentration 0.36, NP type 0.06); (c) global ROS–viability relationship by formulation (Co_3_O_4_ NPs *r* = −0.84; UD–Co_3_O_4_ NPs *r* = −0.93), the steeper slope for UD–Co_3_O_4_ NPs indicating greater cytotoxicity per unit ROS.

## 4. Discussion

The central finding of this study is that green synthesis with *Urtica dioica* extract markedly enhanced, rather than diminished, the anticancer activity of Co_3_O_4_ nanoparticles. Across all three cancer cell lines, UD–Co_3_O_4_ was 5‐ to more than 15‐fold more potent than chemically synthesized bare Co_3_O_4_ on an IC_50_ basis, generated higher intracellular ROS, and, importantly, displayed cancer selectivity (SI > 1) that the bare Co_3_O_4_ lacked. This result is consistent with a growing body of work showing that plant‐mediated cobalt oxide nanoparticles can possess strong, biologically relevant anticancer activity [22, 24, 25, 44], and directly demonstrates that the UD‐mediated formulation exhibits substantially greater anticancer activity than the chemically synthesized bare formulation under the tested conditions.

The elemental and spectroscopic data establish that the two formulations differed in their surface chemistry as intended. The substantial carbon signal seen by EDX only in UD–Co_3_O_4_ (31.14 wt%), together with the shifted O–H band and the emergence of C=C and C–O features in the FTIR spectrum, provides converging evidence for an adsorbed layer of *Urtica dioica* phenolics and flavonoids on the nanoparticle surface. Such phytochemical capping is well documented for green‐synthesized metal and metal‐oxide nanoparticles, where polyphenols act as both reducing and stabilizing agents [20, 23, 45], and *Urtica dioica* extracts in particular are rich in redox‐active constituents [29–31] and have previously been used to generate biologically active nanoparticles [28, 29]. The accompanying increase in hydrodynamic size (108.0 ⟶ 169.9 nm) and polydispersity, and the reduction in the magnitude of the negative zeta potential (−27.23 ⟶ −18.19 mV), are all consistent with such an organic shell partially masking the oxide surface [43]. The redox‐active phytochemicals of *Urtica dioica* leaves are dominated by polyphenols and flavonoids, including chlorogenic and caffeic acid derivatives, quercetin and kaempferol glycosides, together with carotenoids and sterols, as established by comprehensive profiling of nettle extracts [29–31]. These classes of compounds carry the catechol and hydroxyl groups responsible for the O–H, C=C, and C–O signatures observed here, and their high‐resolution identification is now routinely achieved by UHPLC‐Q‐Exactive Orbitrap mass spectrometry, which resolves individual phenolic and flavonoid constituents in plant extracts and links them to measurable antioxidant activity [46]. Beyond acting as reducing and capping agents during synthesis, such adsorbed polyphenols can engage in redox cycling and form polyphenol–metal complexes at the oxide surface, providing a plausible explanation for the enhanced, cancer‐selective activity of the UD–Co_3_O_4_ formulation; direct molecular identification of the coating on the present nanoparticles was, however, beyond the scope of this study and is noted among the limitations below.

The biological data point to oxidative stress as the principal mechanism underlying the enhanced cytotoxicity. ROS generation rose with concentration for both formulations and was tightly anti‐correlated with viability in every cell line, and random‐forest analysis identified ROS as the single most important predictor of viability, ahead of concentration and nanoparticle type. This is fully in keeping with the established redox biology of cancer, in which tumor cells sustain elevated basal ROS and are therefore more vulnerable to additional oxidative insult than normal cells [10, 11, 13]. Cobalt oxide is especially well suited to this mode of action, since its mixed‐valence Co(II)/Co(III) lattice can drive Fenton‐type and Haber–Weiss chemistry and thereby perturb cellular redox homeostasis [14–16]. The behavior observed here therefore fits naturally within the metal‐ion toxicity paradigm, in which redox cycling of a biologically active metal governs both efficacy and toxicity [17, 18].

A key question is why the *Urtica dioica* coating should increase, rather than quench, ROS‐mediated toxicity, given that plant polyphenols are often regarded as antioxidants. Two considerations help reconcile this. First, the ANCOVA showed that UD–Co_3_O_4_ produced significantly more loss of viability per unit of ROS in the cancer lines, but not in L929, indicating that the coating altered the cellular consequences of oxidative stress in a cancer‐selective manner rather than simply generating more ROS indiscriminately. Second, the organic shell increases colloidal dispersion and may enhance cellular interaction and uptake of the nanoparticles, so that more of the redox‐active cobalt oxide is delivered to the intracellular environment, where polyphenol–metal complexes can themselves exhibit pro‐oxidant behavior under the conditions prevailing in tumor cells [5, 47]. A similar enhancement of anticancer activity by phytochemical capping has been reported for other green‐synthesized oxide and bimetallic systems [7, 8, 48]. It should be emphasized that the present dataset establishes a strong association between ROS generation and loss of viability rather than a strict causal relationship. Across all cell lines, ROS was tightly anticorrelated with viability and emerged as the dominant predictor in the random‐forest model, and the ANCOVA showed that UD–Co_3_O_4_ converted a given level of ROS into greater cytotoxicity in the cancer lines specifically; these converging observations are consistent with a causal, ROS‐driven mechanism but do not, on their own, exclude parallel or downstream contributions. Redox‐active cobalt oxide is well placed to act through several interconnected routes: its mixed‐valence Co(II)/Co(III) lattice can drive Fenton‐type chemistry, and cobalt‐based nanomaterials are known to trigger mitochondrial dysfunction, glutathione depletion, lipid peroxidation and ferroptosis‐like death, while intracellular dissolution and cobalt‐ion release provide a further source of oxidative and genotoxic stress [8, 14–16]. The *Urtica dioica* coating is likely to modulate these routes by improving colloidal dispersion and cellular uptake, so that more redox‐active material reaches the intracellular compartment; surface functionalization and colloidal properties are indeed well established determinants of nanoparticle cellular uptake and intracellular cargo delivery [49, 50]. Such amplification of oxidative stress by engineered nanoparticles has been shown to engage regulated cell‐death programs beyond classical apoptosis, including gasdermin‐mediated pyroptosis in arsenic‐trioxide nanoparticle systems [51] and chemodynamic, Fenton‐like ROS generation in tumor‐microenvironment‐responsive metal‐oxide nanoplatforms [52]. A definitive assignment of the operative death pathway for UD–Co_3_O_4_, and the relative contributions of ROS, ion release, uptake and the phytochemical coating, will require orthogonal assays of apoptosis, ferroptosis and mitochondrial function together with ROS‐scavenging controls, which we identify as a priority for future work.

From a translational standpoint, the most significant outcome is the gain in selectivity. Bare Co_3_O_4_ was at least as toxic to the nontumorigenic L929 line as to the cancer lines (SI ≤ 0.28), whereas UD–Co_3_O_4_ achieved SI values above unity in A549 and MDA‐MB‐231. It should be emphasized, however, that the absolute magnitude of this selectivity is modest: a selectivity index of 1.94 in A549 corresponds to only a roughly twofold preference for the cancer line over the nontumorigenic reference, and the value of 1.04 in MDA‐MB‐231 is borderline, indicating essentially comparable sensitivity of the cancer and normal cells in that line. The principal advance reported here is therefore the qualitative shift from a complete lack of cancer selectivity in the bare oxide (SI ≤ 0.28) to a genuine, if still moderate, preferential activity in the green‐synthesized formulation, rather than the achievement of a high selectivity index. Reaching the larger selectivity windows required for therapeutic application would require further optimization of the coating and formulation. Because nanoparticle anticancer activity is meaningful only when accompanied by an acceptable therapeutic window, this improvement is arguably more important than the increase in raw potency.

A more detailed comparison with previously reported Co_3_O_4_ nanoparticles places these results in context across three axes: raw potency, ROS generation, and cancer selectivity. In terms of potency, most green‐synthesized Co_3_O_4_ nanoparticles reported to date show half‐maximal inhibitory concentrations in the mid‐hundreds of micrograms per milliliter; marine‐algae (Grateloupia sparsa) Co_3_O_4_ inhibited HepG2 cells with an IC_50_ of 201.3 μg mL^−1^, Vibrio‐mediated Co_3_O_4_ inhibited CT26 colorectal cells with an IC_50_ of 301.8 μg mL^−1^ at 24 h, and Curcuma longa‐derived Co_3_O_4_ inhibited MDA‐MB‐468 breast cancer cells with an IC_50_ of about 150.8 μg mL^−1^ [15, 24, 53]. Against this background, the IC_50_ values of 29.4–62.6 μg mL^−1^ achieved here by UD–Co_3_O_4_ are several‐fold lower, indicating markedly greater potency, whereas our chemically synthesized bare Co_3_O_4_ (IC_50_ 307 to more than 1000 μg mL^−1^) falls within the range typical of unfunctionalized oxides. With respect to mechanism, the tight ROS–viability coupling reported here is consistent with these earlier studies, several of which attributed Co_3_O_4_ cytotoxicity to ROS generation and oxidative DNA damage [8, 15], but our combined ANCOVA and random‐forest analysis moves beyond a qualitative association by quantifying ROS as the dominant determinant of viability. The clearest contrast, however, is in selectivity: whereas the enhanced potency of UD–Co_3_O_4_ is comparable in magnitude to the best green‐synthesized systems, its cancer selectivity (SI > 1 in two of three lines) is more rarely quantified in the Co_3_O_4_ literature, where selectivity indices relative to a nontumorigenic line are frequently omitted; the few studies that do report normal‐cell controls, such as the Vibrio‐mediated system (IC_50_ 5356 μg mL^−1^ in NIH/3T3 fibroblasts) [24], similarly point to a therapeutic window but do not combine it with the multivariate, ROS‐resolved analysis presented here. The cell‐line–specific pattern, with strongest selectivity in A549, intermediate in MDA‐MB‐231 and weakest in SH‐SY5Y, also indicates that the response depends on the redox and metabolic characteristics of the target cell, consistent with reported heterogeneity in the susceptibility of different tumor types to metal‐oxide nanoparticles [6, 54].

It is worth noting that the primary particle size seen by SEM was considerably smaller than the hydrodynamic size reported by DLS. This is expected: SEM visualizes individual, largely solvent‐free crystallites, whereas DLS reports the hydrodynamic diameter of aggregates together with their hydration and organic shells in suspension. The pronounced aggregation evident in the SEM images, and the elevated polydispersity measured by DLS, both indicate that the particles existed predominantly as aggregates under the assay conditions; this should be borne in mind when interpreting the dose metrics, which are expressed as mass concentrations.

The multivariate approach strengthens the mechanistic interpretation. By combining all cell lines, concentrations, and both formulations into a single model, PCA and random‐forest analysis showed that the dataset is dominated by a single oxidative‐stress axis and that ROS, rather than nanoparticle identity per se, is the proximate determinant of viability. Such data‐driven approaches are increasingly advocated for interpreting complex nanotoxicological datasets and for moving beyond isolated pairwise comparisons [34–36, 55, 56], and their application here provides an internal cross‐check that is consistent with the conventional dose–response and regression analyses. These machine‐learning results should nonetheless be interpreted with appropriate caution, given the modest size of the dataset. Under five‐fold cross‐validation, the random‐forest model showed reasonable predictive performance (*R* ^2^ of about 0.82), which decreased to about 0.72 under the more conservative leave‐one‐cell‐line‐out scheme; this reduction is expected for a dataset of this size and reflects the greater difficulty of generalizing to an entirely unseen cell line rather than an obvious sign of overfitting. Importantly, the ranking of feature importance was stable, with ROS remaining the strongest predictor of viability ahead of concentration and nanoparticle type in every cross‐validation fold. We therefore interpret the feature‐importance results as robust with respect to the relative ordering of predictors, in which ROS dominates, rather than as precise, transferable weight values, and we regard the random‐forest analysis as a confirmatory, hypothesis‐generating complement to the conventional statistics rather than as a predictive tool intended for extrapolation beyond the present experimental space.

This study has limitations. The experiments were conducted in vitro on a small panel of cell lines, and the mass‐based dosing does not distinguish between the effects of primary particle size, aggregation state, and surface coating; the precise molecular identity of the adsorbed phytochemicals was not determined. In addition, the experimental design did not include a parallel treatment group of the *Urtica dioica* extract alone. Therefore, although the UD–Co_3_O_4_ nanoparticles were repeatedly washed and subsequently freeze‐dried before biological testing, a direct biological contribution of plant‐derived components associated with the nanoparticle formulation cannot be formally excluded. The washing procedure was intended to remove unbound and loosely associated extract components, and the EDX and FTIR results provided complementary evidence for the association of plant‐derived organic components with the washed UD–Co_3_O_4_ nanoparticles. Moreover, bare Co_3_O_4_ and UD–Co_3_O_4_ were evaluated under the same biological assay conditions and at the same nanoparticle concentrations, demonstrating a clear difference in their biological responses. However, because the two formulations were produced by different synthetic routes, these differences cannot be attributed exclusively to phytochemical‐mediated surface functionalization. Other physicochemical differences, including particle size, surface charge, and aggregation state, may also contribute to the observed effects. Accordingly, without a parallel extract‐only control, the enhanced ROS generation and cytotoxicity of UD–Co_3_O_4_ are best described as being associated with the UD‐mediated nanoparticle formulation, and the relative contributions of surface‐related physicochemical changes and the intrinsic biological activity of the plant‐derived components themselves cannot be fully separated. The machine‐learning analysis is likewise constrained by the modest dataset, and the feature‐importance scores should be regarded as exploratory and hypothesis‐generating; their generalizability will need to be confirmed on larger and more chemically diverse nanoparticle datasets. The enhanced selectivity and ROS‐mediated mechanism reported here will require confirmation with orthogonal cell‐death and oxidative‐stress assays, dose metrics that account for aggregation, and ultimately in vivo models before any therapeutic relevance can be claimed. Nonetheless, the consistent picture that emerges across elemental, spectroscopic, dose–response, ROS, and multivariate analyses supports the conclusion that *Urtica dioica*‐mediated synthesis is associated with enhanced anticancer activity and altered cancer‐cell selectivity compared with chemically synthesized Co_3_O_4_ nanoparticles, with ROS strongly associated with the observed cytotoxic response.

## 5. Conclusions

In this comparative study, Co_3_O_4_ nanoparticles were prepared by conventional chemical reduction and by green synthesis with *Urtica dioica* leaf extract, and their physicochemical and anticancer properties were evaluated side by side. EDX and FTIR analyses provided complementary evidence for the association of *Urtica dioica*‐derived organic components with the green‐synthesized UD–Co_3_O_4_ nanoparticles, which exhibited increased hydrodynamic size and polydispersity and a reduced magnitude of surface charge relative to bare Co_3_O_4_. Biologically, UD–Co_3_O_4_ showed substantially greater activity than bare Co_3_O_4_: it was several‐fold more cytotoxic against A549, MDA‐MB‐231, and SH‐SY5Y cells, generated higher intracellular ROS, and showed greater cancer‐cell selectivity than bare Co_3_O_4_ based on comparison with the nontumorigenic L929 line.

Convergent evidence from dose–response, ROS, ANCOVA, and machine‐learning analyses indicates a strong association between oxidative stress and the enhanced biological activity, with ROS emerging as the dominant predictor of cell viability in the multivariate analysis and UD–Co_3_O_4_ showing a stronger ROS–cytotoxicity relationship in the tested cancer cells. These findings show that *Urtica dioica*‐mediated synthesis provides an eco‐friendly route and is associated with substantially enhanced anticancer potency and altered cancer‐cell selectivity compared with chemically synthesized Co_3_O_4_ nanoparticles, supporting further investigation of UD–Co_3_O_4_ as a potential ROS‐associated anticancer nanomaterial.

These conclusions should nonetheless be interpreted in light of the study’s main limitations, namely its in vitro design; the use of a single nontumorigenic cell line (L929) as the normal‐cell reference, which is of murine origin and therefore warrants confirmation using normal human cells; the reliance on technical rather than biological replicates; the absence of a parallel *Urtica dioica* extract‐only control, which prevents experimental separation of the intrinsic biological contribution of plant‐derived components from nanoparticle‐associated physicochemical effects; the absence of orthogonal mechanistic validation of the proposed role of ROS in cell death; the limited molecular characterization of the nanoparticle‐associated phytochemical components; and the lack of in vivo evaluation. Future work should include parallel *Urtica dioica* extract‐only controls, confirm the proposed mechanism using orthogonal cell‐death and oxidative‐stress assays, characterize the nanoparticle‐associated phytochemical components in molecular detail, and extend the evaluation to in vivo models to establish therapeutic relevance.

## Funding

The article processing charge (APC) for this publication was financially supported by the Scientific Research Projects Coordination Unit of Dicle University (DÜBAP), Türkiye, under Project No. ZGEF.26.039. The authors also acknowledge the Dicle University Scientific Research Projects Coordination Unit (DÜBAP) for providing laboratory facilities and materials used in this study (Project Nos. ZGEF.24.008, ZGEF.25.010, ZGEF.25.009, and ZGEF.25.008).

## Conflicts of Interest

The authors declare no conflicts of interest.

## Supporting Information

Additional supporting information can be found online in the Supporting Information section.

## Supporting information


**Supporting Information** The supporting information file contains Figure S1 (IC_50_ values and selectivity indices) and Tables S1–S5 (two‐way ANOVA, four‐parameter logistic IC_50_ estimates with confidence intervals, ROS–viability correlations, ANCOVA slope comparison, and random‐forest feature importance).

## Data Availability

The data that support the findings of this study are available from the corresponding author upon reasonable request.
