# Supplementary material for: Co3O4 Nanoparticles Synthesized With Urtica dioica Extract Versus Chemical Reduction: Enhanced ROS Generation, Cytotoxicity, and Cancer‐Cell Selectivity
Source: Bioinorg Chem Appl. 2026 Nov 10;2026:5365097. doi: 10.1155/bca/5365097 (PMC13752486; doi:10.1155/bca/5365097)
Supplement: Supplementary file 1 [file BCA-2026-5365097-s001.docx]

**Supplementary Material**

Co_3_O_4_ nanoparticles synthesized with Urtica dioica extract versus chemical reduction: enhanced ROS generation, cytotoxicity and cancer-cell selectivity

*This file contains the supplementary figure and tables referred to in the main text.*

**Supplementary figure**

**Fig. S1** a IC₅₀ values and b cancer-cell selectivity index (SI) for Co₃O₄ NPs and UD–Co₃O₄ NPs. IC₅₀ values were determined by four-parameter logistic regression on log-transformed concentrations, and SI was calculated as IC₅₀(L929)/IC₅₀(cancer line). For SH-SY5Y the Co₃O₄ IC₅₀ exceeded the tested range (>1000 µg mL⁻¹) and the corresponding SI is not determined (n.d.); the dotted line in b marks SI = 1

**Supplementary tables**

**Table S1** Two-way ANOVA (formulation × concentration) of cell viability for each cell line

| **Cell line** | **Factor** | **F** | **p** | **Sig.** |
| --- | --- | --- | --- | --- |
| A549 | Formulation | 272.6 | <0.0001 | **** |
|  | Concentration | 677.3 | <0.0001 | **** |
|  | Interaction | 32.6 | <0.0001 | **** |
| MDA-MB-231 | Formulation | 237.2 | <0.0001 | **** |
|  | Concentration | 1148.5 | <0.0001 | **** |
|  | Interaction | 41.5 | <0.0001 | **** |
| SH-SY5Y | Formulation | 536.7 | <0.0001 | **** |
|  | Concentration | 318.9 | <0.0001 | **** |
|  | Interaction | 23.3 | <0.0001 | **** |
| L929 | Formulation | 6.8 | 0.0187 | * |
|  | Concentration | 541.5 | <0.0001 | **** |
|  | Interaction | 0.8 | 0.4954 | ns |

**p < 0.05, **p < 0.01, ***p < 0.001, ****p < 0.0001, ns not significant.*

**Table S2** IC₅₀ estimates (four-parameter logistic fit) with 95% confidence intervals, goodness-of-fit and Hill slope

| **Cell line** | **Formulation** | **IC₅₀ (µg mL⁻¹)** | **95% CI** | **R²** | **Hill slope** |
| --- | --- | --- | --- | --- | --- |
| A549 | Co₃O₄ NPs | 320.7 | [221, 465] | 0.968 | 0.40 |
| A549 | UD–Co₃O₄ NPs | 29.4 | [25, 35] | 0.992 | 0.63 |
| MDA-MB-231 | Co₃O₄ NPs | 307.1 | [150, 630] | 0.899 | 0.38 |
| MDA-MB-231 | UD–Co₃O₄ NPs | 55.1 | [47, 65] | 0.993 | 0.56 |
| SH-SY5Y | Co₃O₄ NPs | >1000 | n.d. | 0.976 | 0.46 |
| SH-SY5Y | UD–Co₃O₄ NPs | 62.6 | [33, 118] | 0.908 | 0.36 |
| L929 | Co₃O₄ NPs | 87.3 | [45, 171] | 0.894 | 0.57 |
| L929 | UD–Co₃O₄ NPs | 57.2 | [36, 92] | 0.943 | 0.67 |

*Fits used log₁₀-transformed concentrations with the upper and lower asymptotes constrained to 100% and 0%. n.d. not determined (IC₅₀ > 1000 µg mL⁻¹).*

**Table S3** Pearson correlation coefficients (r) between intracellular ROS generation and cell viability for each cell line and formulation

| **Cell line** | **Co₃O₄ NPs (r)** | **UD–Co₃O₄ NPs (r)** |
| --- | --- | --- |
| A549 | −0.98 | −0.97 |
| MDA-MB-231 | −0.94 | −1.00 |
| SH-SY5Y | −0.97 | −0.95 |
| L929 | −0.92 | −0.90 |

*All correlations were significant (p < 0.001).*

**Table S4** Comparison of ROS–viability regression slopes between Co₃O₄ and UD–Co₃O₄ nanoparticles (ANCOVA)

| **Cell line** | **Slope Co₃O₄** | **Slope UD–Co₃O₄** | **p (slope difference)** | **Sig.** |
| --- | --- | --- | --- | --- |
| A549 | -0.300 | -0.381 | 0.029 | * |
| MDA-MB-231 | -0.299 | -0.409 | 0.005 | ** |
| SH-SY5Y | -0.285 | -0.357 | 0.127 | ns |
| L929 | -0.594 | -0.581 | 0.918 | ns |

*Slopes are the change in cell viability (%) per unit ROS (% of control). *p < 0.05, **p < 0.01, ***p < 0.001, ns not significant.*

**Table S5** Random-forest feature importance scores for prediction of cell viability

| **Feature** | **Importance score** |
| --- | --- |
| ROS | 0.58 |
| Concentration | 0.36 |
| NP type | 0.06 |

*Importance scores were obtained from a random-forest regression model predicting cell viability from concentration, intracellular ROS and nanoparticle type; scores sum to 1.*
